# Biocide Selective TolC-Independent Efflux Pumps in Enterobacteriaceae

**DOI:** 10.1007/s00232-017-9992-8

**Published:** 2017-10-23

**Authors:** Carmine J. Slipski, George G. Zhanel, Denice C. Bay

**Affiliations:** Department of Medical Microbiology and Infectious Diseases, University of Manitoba, Rm 514C Basic Medical Sciences Bldg., 745 Bannatyne Avenue, Winnipeg, MB R3E 0J9 Canada

**Keywords:** Efflux pump, Biocide, Multidrug resistance, Quaternary ammonium compound, Multidrug transporter, Antimicrobial resistance

## Abstract

Bacterial resistance to biocides used as antiseptics, dyes, and disinfectants is a growing concern in food preparation, agricultural, consumer manufacturing, and health care industries, particularly among Gram-negative Enterobacteriaceae, some of the most common community and healthcare-acquired bacterial pathogens. Biocide resistance is frequently associated with antimicrobial cross-resistance leading to reduced activity and efficacy of both antimicrobials and antiseptics. Multidrug resistant efflux pumps represent an important biocide resistance mechanism in Enterobacteriaceae. An assortment of structurally diverse efflux pumps frequently co-exist in these species and confer both unique and overlapping biocide and antimicrobial selectivity. TolC-dependent multicomponent systems that span both the plasma and outer membranes have been shown to confer clinically significant resistance to most antimicrobials including many biocides, however, a growing number of single component TolC-independent multidrug resistant efflux pumps are specifically associated with biocide resistance: small multidrug resistance (SMR), major facilitator superfamily (MFS), multidrug and toxin extruder (MATE), cation diffusion facilitator (CDF), and proteobacterial antimicrobial compound efflux (PACE) families. These efflux systems are a growing concern as they are rapidly spread between members of Enterobacteriaceae on conjugative plasmids and mobile genetic elements, emphasizing their importance to antimicrobial resistance. In this review, we will summarize the known biocide substrates of these efflux pumps, compare their structural relatedness, Enterobacteriaceae distribution, and significance. Knowledge gaps will be highlighted in an effort to unravel the role that these apparent “lone wolves” of the efflux-mediated resistome may offer.

## Introduction

Biocides describe a chemically diverse range of antimicrobial compounds used as antiseptics, disinfectants, and preservatives. In an effort to eradicate potentially infectious bacteria from food preparation, healthcare, and veterinary facilities, biocides such as benzalkonium, chlorhexidine, and triclosan, are commonly used to disinfect exposed surfaces (Gebel et al. 2013), equipment/tubing (Otter et al. 2015), skin, sutures, and wounds (Maillard 2005; Atiyeh et al. 2009). Biocides at high concentrations generally act by disrupting cell envelopes (Maillard 2002; Gilbert and Moore 2005); biocide concentrations (0.1–10% w/v) required for disinfection are too toxic for safe ingestion (orally or parenterally) in most human/animal treatments. Many biocide molecules insert between phospholipid headgroups and displace divalent cations, destabilizing the membrane and reducing osmoregulation (Gilbert and Moore 2005). In contrast to therapeutic antibiotics, biocides have far fewer usage regulations (Levy 2002; Maillard 2005), and are common additives in a wide range of products beyond the healthcare/veterinary setting including: cleansers used in meat/dairy facilities, household products in cosmetics, apparel, wound dressings, and in water/oil pipeline industries to name only a few (McDonnell and Russell 1999; Chapman 2003a; Gilbert and Moore 2005; Atiyeh et al. 2009). Annual biocide usage by commercial, agricultural, and medical industries combined is estimated to reach and exceed annual antimicrobial usage (Van Boeckel et al. 2014), and contributes to contamination of wastewater and soil sediments [as reviewed by (Tezel and Pavlostathis 2011)]. When environmental biocide concentrations accumulate, selective pressure is believed to drive commensal and pathogenic bacterial adaptation and/or acquisition of biocide resistance mechanisms that degrade (Gilbert and McBain 2003) and/or reduce biocide permeability (Levy 2002; Chapman 2003a).

Biocide resistance among Gram-negative Enterobacteriaceae is particularly concerning, since biocide resistance has demonstrated cross-resistance to a variety of antimicrobials (Chapman 2003b; Braoudaki and Hilton 2004; Gnanadhas et al. 2013), including polymyxins (Wand et al. 2017), which may represent a last line of defense. Since Enterobacteriaceae can spread and thrive in wastewater, sewage, and soils beyond their enteric niches (Szmolka and Nagy 2013), biocide adaptation and chronic low-level exposure in these habitats may be promoting antimicrobial cross-resistance (Gaze et al. 2013; Buffet-Bataillon et al. 2016). Enterobacteriaceae are highly adept at acquiring both biocide and antimicrobial resistance genes (Pal et al. 2015), such as extended spectrum beta lactamases (ESBL), through horizontal gene and conjugative element transfer in the gastrointestinal tract (Stecher et al. 2012), or within the environment (Gaze et al. 2005, 2011).

**Fig. 1:**
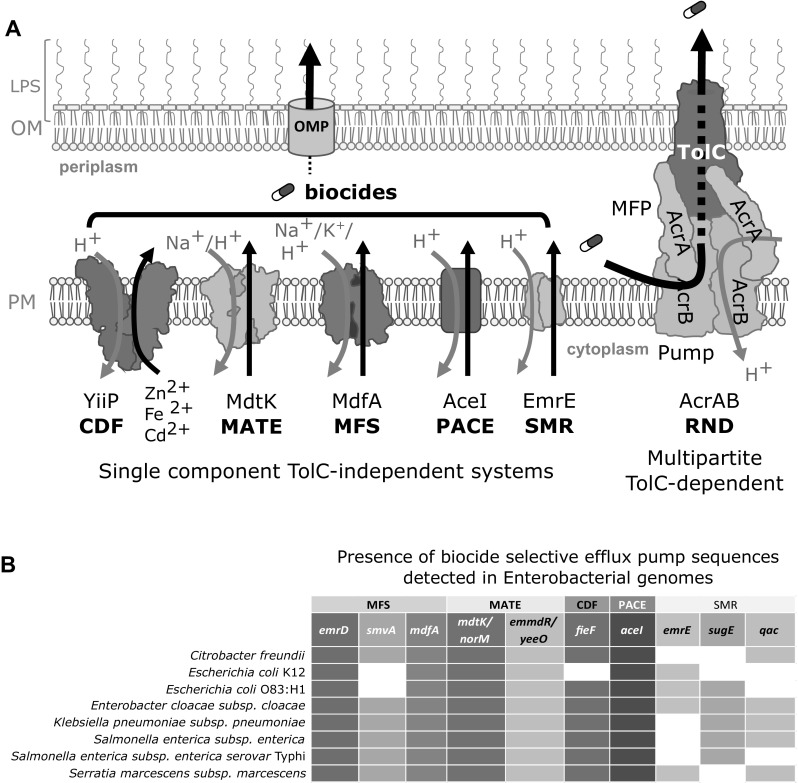
**a** Summary diagram of single component TolC-independent biocide selective efflux pump transporter family members in Enterobacteriaceae. TolC-independent archetypical transporter family members generated from their representative crystal structures for each of the five families: CDF dimer structure CepA/FieF/YiiP (PDB 3H90; Lu et al. 2009), MATE MdtK/NorM (PDB 3MKT; He et al. 2010), MFS MdfA (PDB 4ZOW; Heng et al. 2015), PACE AceI, and SMR EmrE (PDB 3B5D Chen et al. 2007) are shown in the plasma membrane alongside a representative RND multipartite system, AcrA, AcrB, and TolC complex (PDB 5O66; Wang et al. 2017). Gray arrows indicate the direction of ion influx and black arrows show the direction of substrate efflux. **b** A distribution heatmap of TolC-independent efflux pump members within various Enterobacterial genera. Characterized efflux pumps from various Enterobacteriaceae listed in Table 1 were used as query sequences to detect the presence of each member within the completed Enterobacterial genome sequences using tBLASTn (Gertz et al. 2006). The presence (filled squares) and absence (white squares) of efflux pump gene sequences within more than 75% of the listed Enterobacterial species is indicated

By comparison to Gram-positive bacteria, Gram-negative bacteria have higher tolerance to antimicrobials including biocides due to the architecture and composition of their cell envelope that possesses a lipopolysaccharide rich outer membrane, reducing permeability (Fig. 1a). Biocide resistance in Gram-negative Enterobacteriaceae is associated with an assortment of cell envelope alterations that reduce antimicrobial permeability: lipid modifications (Ishikawa et al. 2002; Gilbert and Moore 2005), porin-down regulation (Fernandez and Hancock 2012), outer membrane vesicle formation (Jagannadham and Chattopadhyay 2015; Kulkarni et al. 2015), and intrinsic efflux pump up regulation or efflux pump acquisition (Poole 2002, 2014b; Blair et al. 2014). Among all of these diverse resistance mechanisms, efflux pump activity is a major contributor, particularly in Enterobacteriaceae, due to the presence and diversity of many efflux systems with overlapping (redundant) substrate recognition, and drug polyspecificity. Efflux poses complex resistance challenges to overcome therapeutically, since a single efflux pump can expel a variety of chemically diverse biocides and antimicrobials from the cell. Enterobacterial genomes can encode more than four different efflux pump families on average (Fig. 1b), and many of these families have close homology to eukaryotic transporter protein families (Saier, Jr and Paulsen 2001; Saier et al. 2016), making efflux a formidable resistance mechanism to inhibit (Marquez 2005; Baugh et al. 2014; Opperman and Nguyen 2015).

In *Escherichia coli,* efflux-mediated resistance to antimicrobials is conferred by multipartite protein efflux pump systems that span the outer membrane (OM), periplasm, and plasma membrane (PM) through a protein complex between an outer membrane protein (OMP), a membrane fusion protein (MFP), and efflux pump protein(s) (Nishino et al. 2003). In general, antimicrobial resistance is conferred mainly by the activity of three multipartite efflux pump transporter families; ATP driven ATP-Binding Cassette (ABC) family members such *E. coli* MacAB system (Poole 2014b; Orelle and Jault 2016), by proton motive force driven Resistance-Nodulation-Cell Division (RND) efflux family members AcrAB (Du et al. 2014), and members of the Major Facilitator Superfamily (MFS) such as EmrAB (Kumar et al. 2013b). In Enterobacteriaceae, these systems rely upon an OMP, TolC, to expel various toxic substrates from the periplasmic space across the OM (Zgurskaya et al. 2011).

In addition to TolC-dependent multipartite efflux pump systems, there have been a growing number of single component, TolC-independent, ion/H^+^ driven efflux pump families shown to play a supporting or major role in antimicrobial resistance, most notably to biocides. These single component secondary active efflux pumps can all confer biocide resistance in the absence of TolC and belong to a variety of transporter families; the small multidrug resistance (SMR) family (Bay et al. 2008) part of the drug and metabolite transporter (DMT) superfamily (Jack et al. 2001), multidrug and toxin extrusion (MATE) family (Kuroda and Tsuchiya 2009), major facilitator superfamily (MFS) (Saidijam et al. 2006; Yan 2013), cation diffusion facilitator (CDF) family (Fang et al. 2002; Cubillas et al. 2013), and the recently identified proteobacterial antimicrobial compound efflux (PACE) family (Hassan et al. 2013, 2015b). Hence, they are often referred to as TolC-independent efflux systems (Nishino et al. 2003). It is not well understood if TolC-independent efflux pump members function through a single dedicated, but as yet unidentified OMP(s), or if these efflux systems can utilize a variety of OMPs/channels to completely expel drug substrates from the cell. However, these efflux systems are increasingly important to examine in Enterobacteriaceae based on their ability to confer overlapping substrate specificity, but also resistance to unique substrates not offered by their multipartite system counterparts (Bragg et al. 2014). TolC-independent efflux systems can also expel toxic metabolites and molecules that may be important for cell communication, biofilm formation, and osmoregulation, enhancing their roles in virulence (Piddock 2006; Alcalde-Rico et al. 2016). Efflux pump redundancy and overlapping substrate specificity are some of the major hurdles in elucidating specific efflux pump substrate profiles and in designing improved specific efflux pump inhibitors (Stavri et al. 2007; Tegos et al. 2011). Since many single component efflux pumps are conditionally expressed (Tal and Schuldiner 2009; Hassan et al. 2015a), and are frequently encoded on mobile genetic elements including multidrug resistant plasmids, they are of particular importance to consider in our efforts to combat efflux-mediated multidrug resistance.

Because there have been a number of excellent recent review articles summarizing antimicrobial resistance attributed to multipartite TolC-dependent efflux systems (Poole 2014b; Sun et al. 2014; Li et al. 2015), this article will overview biocide resistance from the perspective of single component, TolC-independent, secondary active efflux pump systems in Enterobacteriaceae, specifically members of the SMR, MFS, MATE, CDF, and PACE families. The aim of this review is to provide an overview of biocides targeted by single component efflux systems, by comparing the biocide and antimicrobial selectivity of characterized members of Enterobacteriaceae, highlight the shared and unique structural features of these pumps, and summarize the significance of their individual activities on resistance and virulence. The knowledge gaps regarding single component efflux pumps conferring biocide resistance will be also discussed in the concluding remarks.

## Biocide Substrates of Single Component Secondary Active Efflux Pumps

**Fig. 2:**
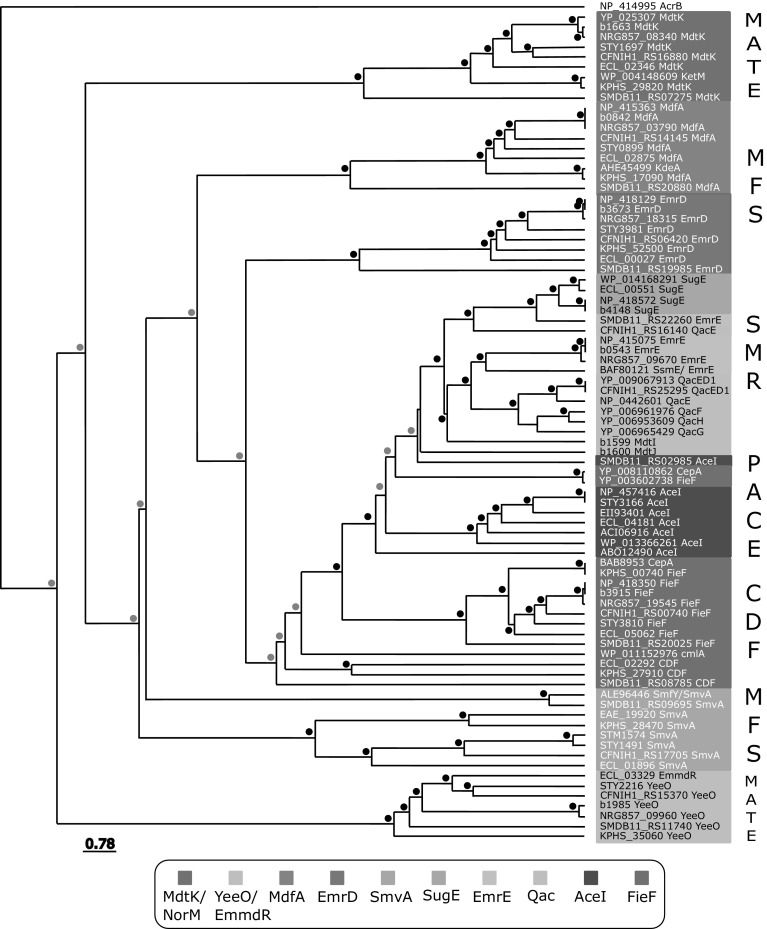
A summary of the average distance percent identities of TolC-independent efflux pump protein family members identified from Enterobacterial genomes. The dendrogram was generated using Jalview software (Waterhouse et al. 2009) based on protein sequence alignments of efflux pump sequences (shown according to GenBank locus tag or protein accession numbers) collected from *E. coli* K12 (b), *E. coli* O83:H1 str. NRG 857C, *Enterobacter cloacae* (ECL), *Salmonella enterica* subsp. *enterica* serovar Typhi str. CT18 (STY), *Citrobacter freundii* CFNIH1, *Klebsiella pneumoniae* subsp. *pneumoniae* (KPHS) species. Protein sequence percent identities were generated from a pairwise alignment was generated using ClustalW BLOSUM weight matrix and bootstrapping of the generated tree matrix was performed using the 'R' statistics software “boot” package (Ripley 2017). Bootstrap *p*-values of < 80% are indicated above each node with a black circle, and *p*-values ranging from 79 to 65% are indicated by gray circles

Single component efflux pumps have garnered criticism regarding their clinical significance and contributions to biocide resistance as well as antimicrobial resistance (Russell 2003; Sheldon Jr. 2005; Hirsch et al. 2011), largely due to their modest minimal inhibitory concentration value increases and lower activity when compared to multipartite efflux systems (Russell 2000; Poole 2002; Hegstad et al. 2010). Identification of specific biocide substrates attributed to specific single component efflux pumps is challenging due to the presence of multiple efflux pump families in any given member of Enterobacteriaceae (Fig. 1b). BLAST-based surveys involving characterized single component efflux members within Enterobacteriaceae genomes reveals that most species possess at least three of the five families discussed herein (Figs. 1b and 2). Single efflux pump gene deletions in Enterobacteriaceae often fail to accurately identify the full range of substrates they may recognize due to the presence of the dominant multicomponent RND system, AcrAB (Sulavik et al. 2001; Tal and Schuldiner 2009). Hence, substrate determination for single component secondary active efflux pumps are commonly determined by pump overexpression in *E. coli* strains lacking *acrB*, such as KAM3 and KAM32 (Chen et al. 2002), to avoid competition with the AcrAB system (Table 1). Single component efflux pumps have been reported to confer resistance to a wide range of chemically diverse biocides and other classes of antimicrobials as shown for MFS and MATE family members (Table 1). In contrast, some pumps demonstrate a preference for one specific class of biocide over others (Table 1). SMR family members confer resistance to a broad range of antiseptics, and relatively few antibiotics (Bay et al. 2008), while CDF member CepA/FieF appears to confer significant resistance to chlorhexidine (Fang et al. 2002) (Table 1). Some of the problems with comprehensive substrate identification are broad range of biocides to include in susceptibility testing and the lack of standard biocide testing methods as noted in a number of reviews over the last two decades (McDonnell and Russell 1999; Gilbert and McBain 2003; Gilbert and Moore 2005; Tumah 2009; Buffet-Bataillon et al. 2012).

**Table 1:** Summary of the antimicrobial resistance conferred by cloned and over-expressed single component efflux systems from various Enterobacteriaceae

Enterobacterial species	Transporter family	Efflux pump	Genbank accession number/locus tag	Biocide resistance (> 2 fold change*)	Antibiotic resistance (> 2 fold change*)	*E. coli* strain used	References
*Citrobacter freundii*	SMR	SugE		MV ET PRO**	ND	HB101	Son et al. (2003)
*Enterobacter cloacae*	MATE	EmmdR/YeeO	ECL_03329	BK ACR ET R6G	NOR CIP LEV TMP ERY FOS	KAM32	He et al. (2011a)
	SMR	SugE	ECWSU1_RS01760; WP_014168291	BK CTAB CTP TPP ET	FOS	KAM32	He et al. (2011b)
	PACE	AceI	Entcl_2273; WP_013366261	BK	ND	BW25113	Hassan et al. (2015b)
*Escherichia coli*	SMR	EmrE	b0543; NP_415075	MV ACR ET CV BK R6G TPP	TMP ERY CHL	KAM3; JM109	Nishino and Yamaguchi (2001)
	SMR	SugE	b4148; NP_418572	CTP CTAB CET	ND	DH5alpha	Chung and Saier Jr. (2002)
	MATE	MdtK/YdhE/NorM	b1663; YP_025307	BK TPP DOC	CHL DOX NOR ENO FOS TMP	KAM3	Nishino and Yamaguchi (2001)
	MFS	EmrD	b3673; NP_418129	SDS BK	NR	KAM3	Nishino and Yamaguchi (2001)
	MFS	MdfA/CmlA/CmlB/Cmr	b0842; NC_000913.3	ACR ET TPP BK R6G	DAU CHL TET CIP NOR DOX TMP ERY TET NEO	HB101; KAM3	Edgar and Bibi (1997a), Nishino and Yamaguchi (2001)
	PACE	AceI	ECTW07793_0407; EII93401	ACR CHX**	ND	TW07793	Hassan et al. (2015b)
*Klebsiella pneumoniae*	CDF	CepA/FieF	BAB89353	CHX	ND	KAM32	Fang et al. (2002), Wand et al. (2017)
	MFS	KdeA/MdfA	KP13_04269; AHE45499	ACR ET TPP	CIP CHL KAN DAU	KAM32	Ping et al. (2007)
	MFS	KmrA/SmvA	KPK_RS11840; WP_012541590.1	MV ET ACR DAPI TPP HOT	NOR GEN ERY	KAM32	Ogawa et al. (2006)
	PACE	AceI	KPK_0842; ACI06916	CHX ACR	ND	BW25113	Hassan et al. (2015b)
	MATE	KetM/MdtK/NorM	KPN_RS10760, WP_004148609	ACR HOT DAPI	CEF NOR CIP	KAM32	Ogawa et al. (2015)
*Serratia marcesens*	SMR	SsmE (EmrE)	BAF80121	ACR CHX ET MV	CIP NOR	KAM32	Minato et al. (2008)
	MFS	SmfY	ABH11_02113; ALE96446	BK ET ACR TPP MV DAPI	NOR	KAM32	Shahcheraghi et al. (2007)
*Salmonella enterica subsp. enterica* Typhimurium	MFS	SmvA (EmrB)	STM1574; NP_460533	ACR MV ET MG PYB CTAB HOT	ERY NOR TET CEF OFL	KAM32; Deletion mutants S. Typhi ATCC14028 s	Santiviago et al. (2002), Ogawa et al. (2006), Villagra et al. (2008), Wand et al. (2017)
	PACE	AceI	STY3166; NP_457416.1	NR	ND	BW25113	Hassan et al. (2015b)
Plasmid/Integron encoded	SMR	QacE	R751p51; NC_001735.4	ACR CV ET PY SO BK BZ CTAB CHX APG		BHB2600; C600	Paulsen et al. (1993), Kazama et al. (1999), Kücken et al. (2000)
	SMR	QacED1	D647_p51049; NC_025134.1	NR	NR	BHB2600; C600	Paulsen et al. (1993), Kazama et al. (1999), Kücken et al. (2000)
	SMR	QacF	D733_p4; YP_006961976.1	CTAB BK	AMP	JM83; Pseudo-monas sp. B13	Ploy et al. (1998); Schluter et al. (2005)
	SMR	QacG	D727_p1037; YP_006965429.1	ND	ND	ND	ND
	SMR	QacH	D616_p71007; YP_006953609.1	ND	ND	ND	ND
	MFS	CmlA2	KPN_RS28175; WP_011152976.1	ND	CHL AMP	JM83	Ploy et al. (1998)
	CDF	CepA/FieF	D647_p28113; YP_008110862.1, ECL_A245; YP_003602738.1	ND	ND	ND	Ren et al. (2010), Berger et al. (2013)

*ND* not determined, *NR* no resistance

Biocide abbreviations: *ACR* acriflavin, *APG* alkylpolyaminoethylglycine hydrochloride, *BK* benzalkonium, *BT BZ* benzethonium, *CET* cetrimide, *CHX* chlorhexidine, *CTAB* cetyltrimethylammonium bromide, *CTP* cetylpyridinium, *DAPI* 4,6-diamino-2-phenylindol, *DOC* doxycholate, *ET* ethidium, *HOT* Hoechst 33342, *MV* methylviologen, *MG* malchite green, *PYB* pyronin B, *PY* pyronin Y, *PRO* proflavin, *R6G* rhodamine 6G, *SO* safranin, *TPP* tetraphenylphosphonium, *TRI* triclosan

Antibiotic abbreviations: *CEF* cefmetazole, *CIP* ciprofloxacin, *CHL* chloramphenicol, *DAU* daunomycin, *DOX* doxorubicin, *ENO* enoxacin, *ERY* erythromycin, *GEN* gentamycin, *FOS* fosfomycin, *LEV* levofloxacin, *NEO* neomycin, *NOR* norfloxacin, OFL oflloxacin, PUR puromycin, *RIF* rifampicin, *TMP* trimethoprim

* Fold change in MIC values determined from wildtype or parental vector containing strain

** 1–1.5 fold change in MIC values determined from wildtype or parental vector containing strain

Antiseptics (benzalkonium, cetrimide, and cetylpyridinium), herbicides (methyl viologen), and dyes (ethidium, acriflavine, and rhodamine 6G) that have one or more permanently charged cationic atoms, typically nitrogen, are referred to as quaternary ammonium compounds (QACs) (Gilbert and Moore 2005; Zhang et al. 2015). QACs appear to be the most common substrate of single component efflux pumps as highlighted in Table 1. In fact, ethidium or Hoechst 33342 are QAC dyes commonly used to identify and validate the activity of most TolC-independent efflux pumps (Blair and Piddock 2016). QACs are also one of the most heavily used biocides with an estimated global usage of 700 kT/year; (Tezel and Pavlostathis 2011), as these compounds are routine additives to commercial, industrial, agricultural, livestock, veterinary, and healthcare products (Zhang et al. 2015).

In contrast, resistance to the bisbiguanide antiseptic chlorhexidine appears to be highly selective and somewhat inconsistent as a substrate for many Enterobacteriaceae efflux pump families, particularly CDF and PACE (Hassan et al. 2015b). Chlorhexidine is commonly added to medical solutions, wipes, baths, and cleansers in medical and veterinary facilities. This apparent bias in substrate selectivity may have as much to do with the inconsistent inclusion of biocide chemicals in routine resistance screening panels, as it does with their over-usage in commercially available products.

It is important to note that the antiseptic triclosan, a chlorinated phenoxyphenol used in oral hygiene products and cleansers, does not appear to be a substrate of single component efflux systems to date (Minato et al. 2008; He et al. 2011a, b). Triclosan is a substrate of multipartite AcrAB systems in *E. coli* (McMurry et al. 1998), and *Salmonella* spp. (Webber et al. 2008) and the unique multipartite system TriABC-OmpH in *Pseudomonas* spp. (Mima et al. 2007). Triclosan resistance is also conferred due to lipid modification; triclosan binds and inhibits the activity of the fatty acid biosynthesis gene *fabI*, an enoyl-acyl carrier protein (Sivaraman et al. 2004). A similar situation is noted for the antiseptic polyhexamethylene biguanide (PMHB), a biguanide used in skin wound care products and ointments, which appears to be a substrate of the multipartite MexCD-OprJ RND system (Fraud et al. 2008).

Cationic biocides like QACs and chlorhexidine function by disrupting the outer and inner plasma membranes of Gram-negative bacilli, denaturing proteins, and enhancing reactive oxygen and nitrogen species generation (Gilbert and Moore 2005). Currently, it remains unclear how efflux pumps can target and expel biocides without succumbing to the effects of the biocide itself, since cationic biocides have the propensity to disrupt membranes at low concentrations and dissipate proton motive force. This phenomenon may explain why many efflux pumps listed in Table 1 confer modest (1-sixfold) increases in biocide MICs. Experiments have demonstrated that the inhibition of efflux systems using electron transport chain disrupting compounds such as CCCP reduce biocide tolerance in Enterobacteriaceae (Braoudaki and Hilton 2005; Rania et al. 2014). Some of the structural features that unify biocide resistance pumps is their increased hydrophobicity and the involvement of negatively charged residues in active site regions of the protein that bind drugs as discussed in the following sections. SMR members have demonstrated a requirement for anionically charged phospholipids, such as cardiolipin and phosphatidyl glycerol (Charalambous et al. 2008; Miller et al. 2009; Dutta et al. 2014), suggesting that the membrane environment may also play an important contribution for biocide recognition and resistance. More studies involving structural and functional analysis of single component efflux pump systems summarized herein will be important in understanding how efflux pumps recognize and transport biocides and other antimicrobial compounds.

## Small Multidrug Resistance (SMR) Efflux Family

**Fig. 3:**
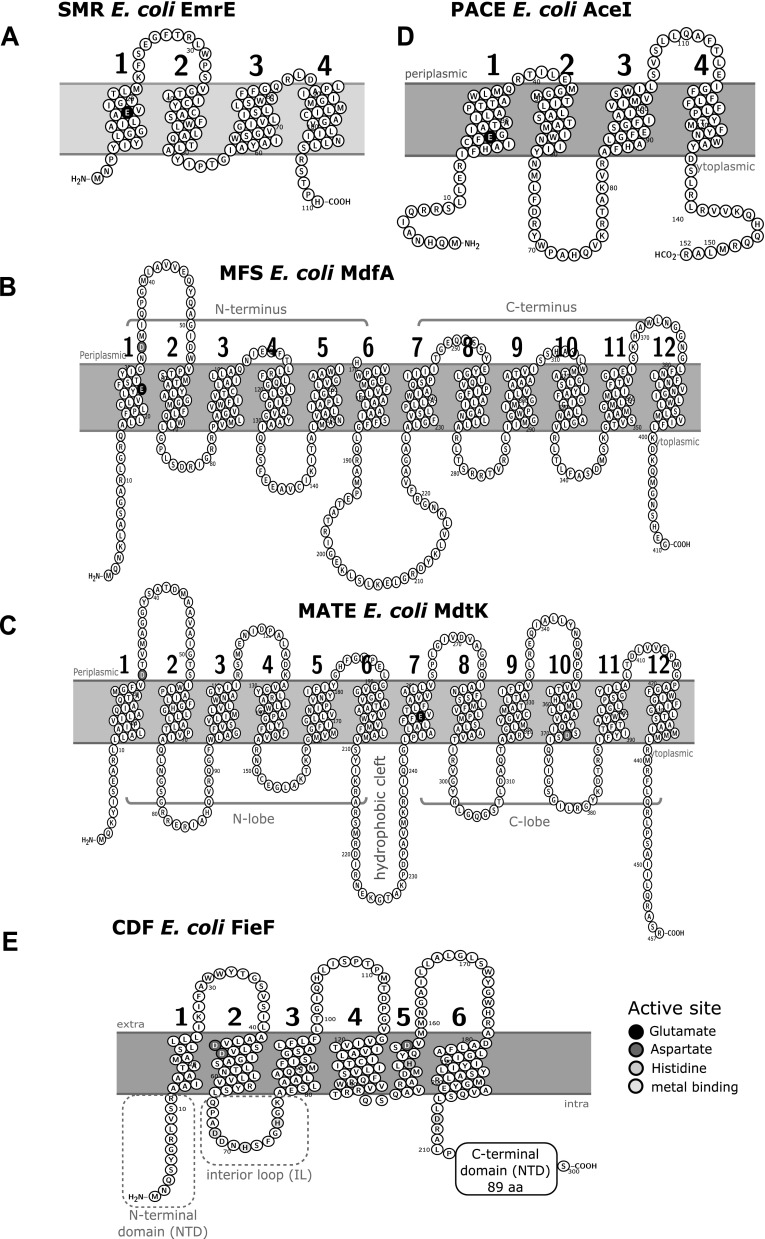
Topology diagrams of biocide resistant efflux pumps representing transporter families SMR, MFS, MATE, CDF, and PACE. **a** Topology diagram of SMR member *E. coli* EmrE (NP_415075); this protein inserts in either orientation, therefore, no membrane orientation is shown. **b** Topology diagram of MFS member *E. coli* MdfA (NP_415363). **c** Topology diagram of MATE member *E. coli* MdtK/NorM/YdhE (YP_025307). **d** Topology diagram of PACE member *E. coli* (EII93401). **e** Topology diagram of CDF member *E. coli* FieF/YiiP (NP_418350). Topology diagrams were generated using the web interface program Protter version 1.0 (Omasits et al. 2014)

SMR protein family members represent one of the smallest efflux families to date, are exclusive to Bacterial and Archaeal kingdoms, and range in length from 100 to 140 amino acids (Bay et al. 2008). SMR genes are inherited vertically on the chromosome, but are frequently encoded by prophages (Wang and Wood 2016), Class 1 and Class 3 integrons (Gaze et al. 2005), and conjugative multidrug resistance plasmids (Bay and Turner 2009) (Table 1; Fig. 1b). The most well characterized and archetypical member of the SMR family is *E. coli* ethidium multidrug resistance protein E (EmrE). At only 110 amino acids in length (12 kDa), EmrE consists of four transmembrane α-helical TMH domains that arrange to form a minimal homodimeric functional unit (Butler et al. 2004; Dutta et al. 2014) (Fig. 3a). The topological arrangement of EmrE monomers within the dimer has been controversial (Schuldiner 2010), as EmrE protein monomers have demonstrated functional activity when both amino and carboxy termini face the same direction (Steiner-Mordoch et al. 2008) or in an asymmetrical arrangement where termini face opposite sides of the membrane (Korkhov and Tate 2009; Morrison et al. 2011; Lloris-Garceras et al. 2012). At the present time, experimental consensus supports an asymmetric SMR dimer topology for the EmrE protein. All SMR members possess a single highly conserved negatively charged glutamate residue (*E. coli* EmrE E14) within the first TMH which participates in both H^+^ and drug binding during transport (Morrison et al. 2015) (Fig. 3a). Despite their small size, SMR members are capable of transporting relatively large cationic compounds, primarily QACs and a limited range of antimicrobials (Table 1), similar to much larger MFS and MATE transporters (12–14 TMH). SMR efflux proteins can be classified into three subgroups, small multidrug protein (SMP); suppressor of *groEL* (SUG); and paired SMR proteins (PSMR) based on phenotypic and phylogenetic sequence analysis (Bay et al. 2008; Bay and Turner 2009, 2016).

The SMP subgroup confers resistance to a wide range of QACs when expressed as a single gene (refer to references provided in Table 1), and SMP members include homologues of *E. coli* EmrE. SMP members may also participate in osmotic regulation, as *E. coli* EmrE overexpression results in hypersaline and pH related loss of growth phenotypes due to the loss of osmoprotectants, specifically betaine and choline (Bay and Turner 2012). In addition to EmrE homologues, SMP subgroup members include integron and multidrug resistant plasmid encoded quaternary ammonium compound resistant proteins (Qac) that also confer resistance to a broad range of QACs (Bay et al. 2010; Buffet-Bataillon et al. 2012). A number of Qac members have been identified in Enterobactericaeae, QacE, QacEΔ1, QacF, QacG, and QacH; where the most frequently identified *qac* gene from multidrug resistant clinical (Kücken et al. 2000; Wang et al. 2008; Pastrana-Carrasco et al. 2012) and food contaminant (Zou et al. 2014; Zhang et al. 2016) genetic surveillance studies is *qacEΔ1*. QacEΔ1 has demonstrated poor QAC efflux activity (30%) in comparison to QacE based on overexpression experiments (Paulsen et al. 1993; Kazama et al. 1999); QacEΔ1 and QacE are nearly identical (up to residue 94), and differ by an in-frame insertion element that disrupts and extends the fourth TMH of QacEΔ1 by an additional five amino acids. It is uncertain why a semi-functional *qacEΔ1* is so highly conserved on 3′ regions of Class 1 integrons when compared to other Qac members including *qacE*. The activity of *qacEΔ1* may improve cross-resistance conferred by its synergy to other conserved resistance genes in the 3′ region including sulfonamide (*sul1*), adenylyltransferase (*aad*), and dihydrofolate reductase (*dfrA*) (Mazel et al. 2000; Su et al. 2006; Gillings et al. 2008); further studies would help clarify its importance. Qac member detection is highly correlated to QAC contaminated areas (Gaze et al. 2011) and serves as a useful marker for QAC biocide pollution (Gaze et al. 2005). Many *qac* genes frequently detected in pathogenic Enterobacteriaceae are also identified from environmentally isolated Enterobacterial biofilms (Gillings et al. 2009), where acquiring one or more Qac members may confer selective advantages such as an ability to expel toxic and QAC-based metabolic compounds as demonstrated by other SMR members.

Members of the SUG subclass are phylogenetically distinct from SMP members (Bay and Turner 2009), and were originally named for their ability to suppress *groEL* chaperonin protein E (SugE) in *E. coli* that was later demonstrated to be a cloning artifact (Bishop and Weiner 1993; Bishop et al. 1995). Characterized SUG members include SugE from *E. coli* and *Citrobacter freundii*, where *E. coli* SugE members have demonstrated resistance to a narrow range of long acylated QACs, including cetrimide and cetylpyridinium (Chung and Saier Jr. 2002). Resistance to other metal containing biocides has also been demonstrated by Gram-negative *Aeromonas molluscorum* SugE homologs that specifically efflux di- and tri-butyltin (Cruz et al. 2013); tin containing biocides banned from use in developed countries since the 1990s. Mutational studies of *C. freundii* Sug members can demonstrate function as an importer as well as an exporter (Son et al. 2003), similar to EmrE (Brill et al. 2012). A recent biofilm study of *E. coli* single gene deletion mutants identified that a *sugE* deletion enhanced biofilm biomass formation, suggesting that under wildtype conditions this pump may expel metabolites regulating biofilm formation (Bay et al. 2017). Although SUG members are inherited chromosomally, many Enterobacteriaceae SugE homologs are detected from mobile genetic elements carried on multidrug resistant plasmids, particularly in the poultry industry (Chung and Saier Jr. 2002; Hegde et al. 2016). This suggests that SMR members, such as SUG and SMP Qac proteins, may be selectively enriched either on specific mobile genetic elements or by QAC pollution (Gaze et al. 2005).

The PSMR subclass differs from SMP and SUG subgroups due to the expression of two distinct genes to confer QAC resistance forming a functional heterodimer (Bay et al. 2008). Phylogenetic analysis of PSMR subgroup homologues indicates that these proteins evolved more recently due to gene duplications from SMP and SUG group members, however, in Enterobacteriaceae PSMR members share close homology to *E. coli* MdtIJ (YdgEF) related to the SMP subclass (Bay and Turner 2009). By comparison to EmrE, PSMR proteins have longer hydrophobic loops between TMH1-4 and an extended carboxyl-terminus (Kikukawa et al. 2007), that fixes their topology and multimerization into asymmetric heterodimers (Drew et al. 2002; Rapp et al. 2004). While MdtIJ does confer multidrug resistance to QACs and cationic dyes, they also contribute towards polyamine resistance indicating they may play an important role in preventing polyamine toxicity (Higashi et al. 2008).

## Major Facilitator Superfamily (MFS) Efflux Family

MFS transporters span all three kingdoms of life and currently form 74 families (Reddy et al. 2012). In Enterobacteriaceae, characterized MFS members that are capable of conferring biocide resistance include the TolC-dependent multipartite efflux pumps systems; *E. coli* EmrKY and EmrAB (Li and Nikaido 2004; Tanabe et al. 2009), and TolC-independent as well as monomeric TolC-independent efflux pumps: EmrD (Naroditskaya et al. 1993; Nishino and Yamaguchi 2001; Yin 2006), and MdfA/CmlA/Cmr (Edgar and Bibi 1997a; Bohn and Bouloc 1998) (Table 1; Figs. 1b, 2). Enterobacteriaceae typically encode two or three different TolC-independent biocide selective MFS members that confer both QAC and fluoroquinolone resistance: EmrD, MdfA, and SmvA (Table 1, Figs. 1b, 2). The third MFS family member known as SmvA/KmvA/SmfY is distinguished for its ability to confer methyl viologen resistance and has been functionally characterized from *Salmonella enterica* serovar Typhimurim (Hongo et al. 1994; Santiviago et al. 2002; Villagra et al. 2008), *Serratia marcescens* (Shahcheraghi et al. 2007) and *K. pneumoniae* (Ogawa et al. 2006) species (Table 1).

MFS members typically range from 400-600 amino acids in length and possess 12 TMH that form two 6 TMH domains at the N- and C- termini (Yan 2013). TolC-independent MFS members from Enterobacteriaceae share close identity to either *E. coli* EmrD, SmvA, or MdfA as shown in Fig. 2. Enterobacteriaceae monomeric biocide selective efflux pumps belonging to the MFS members generally possess 12 TMH (Fig. 3b), with the exception of *Serratia marcescens* SmfY (Shahcheraghi et al. 2007) with 14 TMH as noted by (Hofmann and Stoffel 1993; Krogh et al. 2001) and predicted by TMpred and TMHMM. Regardless of TMH number, both N- and C-termini of MSF generally face the cytosolic side of the membrane, and structural similarity between each N- and C-terminal domain suggests that the MFS efflux protein originated from a gene duplication event (Saier Jr. 2001; Saier Jr. and Paulsen 2001; Reddy et al. 2012). Monomeric MFS transporters function through two pseudosymmetrical 6 TMH domain units that surround a central hydrophobic substrate binding pocket with fluctuating drug/ion access via a rocker-switch mechanism (Yan 2013; Quistgaard et al. 2016) (Fig. 3b). Most MFS members contain two highly conserved, negatively charged residues in their central substrate binding pocket; in *E. coli* MdfA the loss of residues E26 and D34 in TMH1 eliminates drug efflux activity (Adler et al. 2004; Sigal et al. 2006). However, in a previous study a pair of glutamates required for drug efflux activity could be removed and replaced in different TMH (Sigal et al. 2009), highlighting the conformational plasticity of these proteins (Fig. 3b). Some studies demonstrate that MFS transporters may utilize Na^+^/K+ as well as H^+^ in drug antiport function (Edgar and Bibi 1997a; Mine et al. 1998; Lewinson et al. 2004). The ability to use Na^+^ and H^+^ appears to confer an osmoregulatory function for these pumps, as observed for alkali tolerant *E. coli* MdfA and *K. pneumoniae* KdeA/MdfA strains when these pumps are over-expressed (Lewinson et al. 2004; Ping et al. 2007). Alkali tolerance synergizes well with known cationic biocide resistance mechanisms as both are stress induced responses in Enterobacteriaceae [as reviewed by (Rowbury 2003; Poole 2014a; Buffet-Bataillon et al. 2016)].

TolC independent MFS members can be inherited vertically on the chromosome, as demonstrated by *E. coli* MdfA and EmrD (Edgar and Bibi 1997b; Nishino and Yamaguchi 2001), as well as on mobile genetic elements such as Cmr/CmlA, which are found on integrons and multidrug resistant conjugative plasmids making them highly transmissible, particularly within environments highly contaminated by QACs (Bohn and Bouloc 1998; Ploy et al. 1998; Heuer et al. 2004; Bischoff et al. 2005). In *E. coli*, MFS efflux proteins make up about half of all unique efflux transporters known, highlighting their importance for biocide and multidrug resistance (Kumar et al. 2013a). Experiments involving *E. coli* RND AcrAB pump deletions demonstrated that chromosomally encoded SMR and MFS members, EmrE and MdfA respectively, were important to confer antimicrobial resistance under specific growth conditions (Tal and Schuldiner 2009), underscoring their importance in expelling substrates, specifically methyl viologen, a biocide not recognized by RND pumps (Villagra et al. 2008). Expression and deletion of *E. coli* MFS member MdfA has also demonstrated its involvement in enhancement of biofilm formation (Matsumura et al. 2011; Soto 2013b), suggesting it may play a role in biofilm establishment and biocide tolerance. Hence, with multiple copies of TolC-independent MFS members in a single Enterobacteriaceae species, the contributions of these pumps to biocide resistance may be highly important when conditions arise that prohibit RND and other multipartite system activities.

## Multidrug and Toxic Compound Extrusion (MATE) Efflux Family

MATE protein family members are part of the larger multidrug/oligosaccharidyl-lipid/polysaccharide (MOP) superfamily (Hvorup et al. 2003). MATE family members are currently subdivided into three main subfamilies according to kingdom and sequence phylogeny: Family 1 bacterial MATEs; Family 2, eukaryotic MATEs subdivided as 2A fungal, 2B plant, 2C animal, and 2D protozoan; and Family 3, bacterial and archaebacterial MATEs (Omote et al. 2006; Kuroda and Tsuchiya 2009). The first bacterial MATE member, NorM, is related to family 1 and was identified from *Vibrio parahaemolyticus* (Morita et al. 1998), and has high sequence similarity and homology to MdtK (YdhE) based on its identification in *E. coli* (Figs. 1b and 2). There is no single archetypical MATE member to date due to the low sequence identity between MATE homologues (~ 40%), which can range in length from 440 to 500 amino acids (Omote et al. 2006). However, NorM crystal structures from *Neisseria gonorrhea* (Lu et al. 2013) and *Vibrio cholerae* (He et al. 2010) best represent bacterial MATE transporters at present. Functional Na^+^ dependency has not been demonstrated for all MATE families (Mishra and Daniels 2013), but bacterial NorM homologues have demonstrated both Na^+^ and H^+^ dependence (Lu et al. 2013; Jin et al. 2014). MATE transporters typically arrange into 12 TMH (Fig. 3c) based on *V. cholerae and N. gonorrhe*a NorM crystal structures, forming two lobes at the N- (TMH 1-6) and C- (7-12) terminal domains with a hydrophobic cleft connected by a cytoplasmic loop between TMH 6–7 similar to MFS members (as reviewed by (Kuroda and Tsuchiya 2009; Lu et al. 2013; Du et al. 2015)). Based on the conservation and symmetry within the N- and C-lobes, each domain within MATE proteins likely arose due to gene duplication or fusion event similar to MFS members (Lu et al. 2013). However, a 13th TMH may be present at the C-terminus as suggested by an epitope tagged eukaryotic MATE1 homologue (Zhang and Wright 2009) and hydropathy plots. Similar to SMR and MFS members, functionally essential and highly conserved negatively charged residues serve as active sites for binding cationic drugs and/or ions (Kuroda and Tsuchiya 2009; Du et al. 2015). Negatively charged residues are positioned in both lobes at TMH1 D32, TMH7 E252, and TMH10 D368 (Omote et al. 2006) numbered according to *E. coli* MdtK (Fig. 2). MATE efflux proteins demonstrate a rotational symmetry within the first and last TMH loops forming the N and C lobe domains respectively, and a cytoplasmic loop connects the two halves between TMH loops 6 and 7 (Fig. 2) (He et al. 2010; Nishima et al. 2014). Substrate binding and release has been shown to function through an inward and outward alternating conformer known as a rocker-switch model (Nishima et al. 2014). The hydrophobicity of the drug binding clefts and highly conserved negatively charged residues share similar characteristics with other cationic biocide selective transporter families.

Cloned and characterized Enterobacterial MATE members include *E. coli* MdtK (Morita et al. 1992), *Klebsiella pneumoniae* KetM/MdtK (Ogawa et al. 2015), and *Enterobacter cloacae* EmmdR (He et al. 2011a), which all confer resistance to QAC antiseptics and DNA intercalating dyes as well as quinolone antimicrobials (Table 1) driven by Na^+^ and/or H^+^ motive force similar to NorM (Jin et al. 2014). *E. cloacae* EmmdR shows much higher sequence identity to *E. coli* MATE member YeeO than MdtK (Fig. 2), suggesting that two different biocide selective TolC-independent MATE members are present in Enterobacteriaceae. In Enterobacteriaceae, MATE genes are encoded chromosomally and have not been confidently identified from multidrug resistant plasmids yet according to BacNet (Pal et al. 2014) and GenBank searches); genes encoding MATE members often possess an alternative start codon, GTG, that may play a regulatory role in reducing its expression indicative of conditional regulation (Long et al. 2008; Ogawa et al. 2015). MATE homologue *E. coli* MdtK overexpression has been shown to rescue 8-oxoguanine-repair-deficient hypermutator phenotypes and protect cells against H_2_O_2_ damage, suggesting that NorM homologues may act as a guanine oxidation backup system when toxic metabolic reactive oxygen species build up (Guelfo et al. 2010). Since QAC biocides have demonstrated an ability to induce significant oxidative damage to cells, and many have complex chemical structures reminiscent of fluoroquinolones (as reviewed by (Buffet-Bataillon et al. 2016)), MATE protein biocide efflux may be a coincidental by-product related to oxidative stress compounds rather than specific biocide resistance. In *E. coli,* MdtK and RND pump AcrAB were both shown to influence cell growth during stationary phase (Yang et al. 2006), potentially due to the secretion of metabolic quorum sensing molecules such as 4-quinolone that may influence cell–cell communication and biofilm formation (Nair et al. 2016). The fact that MATE members function as sodium-drug antiporters and regulate quorum sensing factor release, and confer protection against reactive oxygen species, suggests that their role is multifaceted and involved in protection and regulating cell metabolism, in addition to multidrug resistance.

## Proteobacterial Antimicrobial Compound Efflux (PACE) Family

The newest addition to the efflux transporter proteins is the PACE family, which was discovered in *Acinetobacter baumannii* by Hassan et al. 2013, and named accordingly as Acinetobacter chlorhexidine efflux protein I (AceI) (Hassan et al. 2015b). As the name suggests, the PACE efflux proteins have specificity for chlorhexidine as well as other cationic membrane disrupting QAC antimicrobials such as, benzalkonium chloride and acriflavine (Table 1) (Hassan et al. 2015b). *A. baumannii* AceI efflux pumps were discovered from chlorhexidine shock induction experiments monitoring transcriptionally upregulated genes; chlorhexidine was selected for its frequent antiseptic usage in industrial and hospital settings (Hassan et al. 2013). PACE efflux pumps are predicted to function as a secondary active drug/H^+^ antiporter (Hassan et al. 2015b). Secondary structure predictions of AceI and its homologues indicate that these proteins are short in length (180 amino acids) and have two tandem bacterial transmembrane pair (BTP) domains (Fig. 3d) (Hassan et al. 2015a). AceI homologues also rely upon a negatively charged glutamate (*A. baumannii* E50; *E. coli* E22) residue in the first TMH domain (Fig. 2), which was shown to inhibit transport activity, but not drug binding, suggesting other as yet unidentified residues may be involved in chlorhexidine binding (Hassan et al. 2013). Although PACE family members are primarily identified from y-proteobacteria, they are not exclusively found in proteobacteria, there are also a few homologous members in the firmicutes *Veillonella parvula,* and likely in representatives of other phyla (Hassan et al. 2015a). In *E. coli,* there is an AceI homolog identified in the strains TW07793 and KTE84, which are flanked by transposase and phage insertion sequences, suggesting these PACE transporters were acquired laterally from related proteobacteria (Hassan et al. 2013). To date, PACE efflux proteins have only been identified in the domain Eubacteria demonstrating similarity to the SMR family of transporters. PACE family members may also be regulated by a general stress response mechanism (Hassan et al. 2013), but it remains unclear how similar the stress response is to other single component transporter families.

## Cation Diffusion Facilitator (CDF) Superfamily

Cation Diffusion Facilitators (CDFs) represent a family of transporters in all three kingdoms of life that confer metal tolerance/resistance by efflux of zinc and heavy metal ions (Cubillas et al. 2013; Kolaj-Robin et al. 2015). Phylogenomic groupings of prokaryotic CDF family members are based on metal ion specificity (Cubillas et al. 2013), and thus far only one CDF member has demonstrated biocide resistance, *K. pneumoniae* chlorhexidine efflux protein A (CepA) (Fang et al. 2002), also annotated as ferrous iron efflux protein F (FieF/YiiP) due to its ability to relieve toxic concentrations of iron stress (Grass et al. 2005). The archetypical structural arrangement of the bacterial CDF protein family is based on the crystal structures of the zinc binding CDF member in *E. coli* FieF/YiiP (Lu and Fu 2007; Lu et al. 2009). *E. coli* FieF/YiiP forms a functional homodimer where each monomer has a modular two domain architecture in which both TMH domains form a hydrophobic cleft, and cytoplasmically exposed domains form a metal ion binding domain (Fig. 2) (Wei et al. 2004). Monomeric CDF protein arranges to form 6 TMH with three domains: an N-terminal domain (NTD), histidine rich interconnecting loops (ILs), and 100 a.a. long C-terminal domain (CTD) arranging together to form a TMH and cytoplasmic domain that coordinates the metal ion transport from the cytoplasm (Fig. 3e) (Kolaj-Robin et al. 2015). Dimer stabilization and metal ion coordination is accomplished by conserved aspartate and histidine residues within the TMH domain of the *E. coli* FieF protein, TMH2 (D45, D49), and TM5 (H153, D157); as well as a number of hydrophobic cleft forming residues located in TMH2, TMH3, and TMH5; along with salt bridge forming aspartate and lysine residues located in TMH3 and cytoplasmic domains (Wei and Fu 2006; Fu 2010; Kolaj-Robin et al. 2015). Based on current structures of the *E. coli* YiiP (Lu and Fu 2007; Coudray et al. 2013), it is uncertain how chlorhexidine transport is accomplished by *K. pneumoniae* CepA, despite their high sequence identity (86%) (Fang et al. 2002); nor is it clear if other FieF homologues can confer biocide resistance. FieF homologues are well represented in most pathogenic Enterobacterial species (Fig. 1b), suggesting that metal and chlorhexidine efflux may be important for pathogenicity. Two transport mechanism models are currently being validated (Kolaj-Robin et al. 2015). *cepA* genes are frequently detected in biocide resistant *Klebseilla* spp. isolates (Abuzaid et al. 2012; Naparstek et al. 2012; Rania et al. 2014), as well as in *Enterobacter* sp. (Ren et al. 2010) either chromosomally (Cubillas et al. 2013), or from multidrug resistant plasmids (Rania et al. 2014). *cepA* is commonly associated with other biocide selective efflux pump family members, most frequently SMR family *qac* genes (Azadpour et al. 2015), and/or MFS *cmlA* members (Naparstek et al. 2012). The association of toxic metal efflux pumps with efflux pumps conferring biocide resistance is not uncommon on mobile genetic elements (Gullberg et al. 2014; Wales and Davies 2015; Pal et al. 2015), as both toxic/heavy metals and biocides are used together as antiseptics in livestock and healthcare settings. Further examination of the potential biocide resistance conferred by FieF homologues will hopefully provide additional insights into the role they play in virulence, and antimicrobial/metal resistance in Enterobacteriaceae.

## Knowledge Gaps

Many questions remain regarding the structure, function, and regulation of TolC-independent efflux pumps. Regarding functional considerations, how biocides are completely expelled from within the periplasm after single component efflux activity remains unclear. Do these pumps function with one or more multipartite systems, such as the AcrAB-TolC system as suggested from an *E. coli* study of combinatorial RND AcrAB, SMR *emrE,* and MFS *mdfA* gene deletions (Tal and Schuldiner 2009)? Or is the expulsion of substrates across the outer membrane from the periplasm reliant on passive diffusion through one or more OMP? Evidence supporting specific OMP involvement has been demonstrated for SMR member EmrE, which demonstrated an association with OmpW to expel osmoprotectants and methyl viologen (Beketskaia et al. 2014). What roles do these efflux systems contribute to biofilms? QACs are frequently used to eradicate biofilms (McBain et al. 2004; Buffet-Bataillon et al. 2011), the presence of QAC resistant TolC-independent efflux pumps may indicate that these systems play a role in the establishment and maintenance of biofilm growth (Russell 2003; McBain et al. 2004; Houari and Di Martino 2007; Buffet-Bataillon et al. 2011; Soto 2013a). Therefore, single component efflux pumps may contribute to biofilm formations that are more difficult to treat in healthcare and industrial settings, making this an imperative area for future study.

The association of lipid modifications caused by biocide exposure and upregulated efflux activity is also present in biocide resistant Enterobacteriaceae. Most biocide resistance studies examine laboratory adapted strains that exhibit changes not only in efflux pump activity but also in lipid modifications which alter head group charge and fatty acid saturation (Braoudaki and Hilton 2004, 2005; Langsrud et al. 2004; Bore et al. 2007). Studies of SMR member EmrE suggest that efflux pump activity is regulated by lipids, specifically low abundance anionic phospholipid cardiolipin (Charalambous et al. 2008; Miller et al. 2009; Dutta et al. 2014). Understanding the interconnectedness of biocides, lipids, and efflux pump proteins together, may help improve the design of specific efflux pump inhibitors.

With regards to function, the biocide concentrations that individual efflux pumps can confer resistance to may differ depending on how the cells are grown: the physiology of the cells (planktonic versus surface attached biofilm), the type of growth media used, and the background genotype of the bacterial strain used to measure minimum inhibitory concentration values attributed to specific pump activity [as reviewed by (Baugh et al. 2014; Blair and Piddock 2016)]. *E. coli,* being a reservoir for mobile genes in the environment (Szmolka and Nagy 2013), and a representative member of Enterobacteriaceae, make it an ideal candidate to study single component efflux pumps as a model organism. Considering many TolC-independent efflux pumps exhibit conditional activity that influences osmoregulation (SMR, MFS, and MATE), DNA repair (MATE), metal transport (CDF), and metabolite regulation (SMR, MFS, and MATE), these efflux systems likely have overlooked importance. Further insights into the nature of the stress response(s) involved in single component efflux system expression beyond standard laboratory growth conditions may help provide greater insights into conditional phenotypes conferred by these efflux pumps.

## Concluding Remarks

The biocide selective transporter families focused on in this review are found in many pathogenic Gram-negative Enterobacteriaceae, and the fact that they may confer cross-resistance to other antimicrobials, highlights their clinical importance (Table 1). In addition to drug resistance, many of these TolC-independent efflux pumps contribute towards other physiological processes such as osmoregulation (SMR, MFS, and MATE), biofilms (SMR, MFS, and MATE), quorum sensing (MATE), tolerance to toxic metals (CDF) and reactive oxygen species (MATE); these features demonstrate the adaptability, and multifaceted function that are hallmarks of single component efflux pumps.

Single component efflux pump systems have been largely overlooked as compared to other well studied efflux families, such as multipartite RND pumps, however, their prevalence on mobile genetic elements, along with their frequent detection in healthcare and environmental Enterobacterial isolates, suggests that there is sufficient selective pressure to maintain and spread these efflux pumps to other systems. The selection for these single component efflux pumps may be perpetuated by the widespread overuse of antimicrobials, especially cationic antiseptics such as QACs, and the detection of these efflux pumps may be a marker for anthropogenic contamination of the environment with these antimicrobials; driving a shift towards decreased membrane permeability in Gram-negative bacilli. The recent ban of biocides from hand soaps by the US Food and Drug Administration (Sept. 2016), which includes triclosan, and many QAC antiseptics as well as even more stringent regulations imposed on the use and inclusion of biocides in commercial products by the European union over the last 10 years (Biocidal Products Regulation; https://ec.europa.eu/health/biocides/policy_en), provide some hope that more responsible biocide regulations and stewardship may soon be on the global horizon. By reducing environmental biocide exposure, we may begin to reduce the selective pressure driving the spread and adaptation of these efflux systems towards biocides and antimicrobials.
